# Incidence of Opioid Use Before and After Parkinson's Disease Diagnosis

**DOI:** 10.1002/ejp.70056

**Published:** 2025-06-09

**Authors:** Majd Al‐Sagheer, Niina Karttunen, Anne Paakinaho, Marjaana Koponen, Valtteri Kaasinen, Sirpa Hartikainen, Miia Tiihonen, Anna‐Maija Tolppanen

**Affiliations:** ^1^ School of Pharmacy University of Eastern Finland Kuopio Finland; ^2^ Centre for Medicine Use and Safety, Faculty of Pharmacy and Pharmaceutical Sciences Monash University Parkville Victoria Australia; ^3^ Clinical Neurosciences University of Turku Turku Finland; ^4^ Neurocenter Turku University Hospital Turku Finland

## Abstract

**Background:**

Pain is a common symptom of Parkinson's disease (PD). It occurs also as a prodromal sign of PD. It has not yet been described how the use of analgesics changes over time in persons with PD. We investigated the incidence of opioid use from 5 years before to 5 years after PD diagnosis and compared the incidence to a matched cohort.

**Methods:**

This study included 15,763 people diagnosed with incident PD in 2001–2014 and 62,907 matched comparison persons without PD from the Finnish nationwide register‐based study on Parkinson's disease (FINPARK). Initiation rates of opioid use during the follow‐up were calculated in 6‐month time windows, and the difference between persons with and without PD was described using incidence rate ratios (IRRs).

**Results:**

Opioid initiation was more common among persons with PD (37.0%) compared to people without PD (31.2%). The difference between the groups emerged 3 years before the PD diagnosis IRR 1.33 (1.16–1.53), and remained until the end of the follow‐up. Mild opioids, such as tramadol and codeine, were the most commonly initiated opioids, and the largest difference in their use was observed 6 months prior to the diagnosis date, while differences in strong opioids emerged after the PD diagnosis. Initiation rates increased over time and remained above those of the comparison group after the PD diagnosis for all opioid classes.

**Conclusions:**

The findings demonstrate the incidence of moderate/severe pain in PD, which requires treatment with opioid‐class analgesics. Further studies are needed to understand the long‐term impact of opioid use in persons with PD.

**Significance Statement:**

Initiation rate of opioids is increased in persons with Parkinson's disease already at premotor or early motor stage, before the diagnosis is confirmed. Shift towards stronger opioids is observed over the disease progress. Further studies are needed to investigate optimized pain management strategies in Parkinson's disease.

## 1 Introduction

Although the diagnosis of Parkinson's disease (PD) is based on motor symptoms, nonmotor symptoms are common, often preceding the motor symptoms (Postuma et al. 2015). Pain is a significant non‐motor symptom of PD (Defazio et al. 2008), prevalence ranging between 40% and 85% (Broen et al. 2012). It can occur at any stage of PD, already years before the diagnosis (Bohlken et al. 2022; Schrag et al. 2015; Simonet et al. 2022). In a study of 402 PD patients, 22% of patients with dystonic pain and 25% of those with nondystonic pain reported the onset of pain before antiparkinsonian therapy (Defazio et al. 2008).

Different classifications for pain in PD have been proposed (de Andrade et al. 2023). The Ford classification categorised pain in PD into musculoskeletal, neuropathic, dystonic, akathisia, and central pain (Ford 2010). Recently, a mechanistic classification system of pain in PD, differentiating PD‐related pain, was proposed (Mylius, Perez Lloret, et al. 2021). It categorises PD‐related pain as nociceptive, neuropathic, and nociplastic. In a cohort study of 159 patients with PD, 77% had PD‐related pain, and 22% had pain unrelated to PD (Mylius, Perez Lloret, et al. 2021). Altogether, 15% of those with PD‐related pain had more than one type of pain (Mylius, Perez Lloret, et al. 2021).

The clinical approach to treating pain in PD depends on the pain type and mechanism (Seppi et al. 2019; Sophie and Ford 2012). There are no PD‐specific guidelines, but adjustment of the dopaminergic medication is the initial step in pharmacological treatment (Seppi et al. 2019). Analgesics, such as opioids, non‐steroidal anti‐inflammatory drugs (NSAIDs) and paracetamol, can be prescribed (Buhmann et al. 2020). Although oxycodone was considered as potentially efficacious in relieving PD pain in one trial (Trenkwalder et al. (2015)), and there are recommendations concerning prolonged release oxycodone‐naloxone for chronic pain in PD (Mylius, Möller, et al. 2021), the efficacy evidence is currently insufficient (Brefel‐Courbon et al. 2024; Seppi et al. 2019). Other opioids can also be used in managing chronic pain in PD (Edinoff et al. 2020). Opioids need to be prescribed cautiously due to their adverse effects and events (Guerriero 2017).

Most previous studies on opioid use in PD have been small and cross‐sectional. In a study of 181 PD patients, 9% of patients who used analgesics used opioid derivatives (Buhmann et al. 2017). Two surveys assessed the prevalence of analgesics according to the World Health Organisation's analgesic ladder (WHO 2018). In a survey of 123 patients, 25.2% used weak opioids (Lee et al. 2006.) In another survey of 450 patients with PD, 9.6% of those with PD pain and 15.3% with non‐PD pain had used weak opioids (Nègre‐Pagès et al. 2008). A large‐scale cross‐sectional study based on French administrative databases reported that 38.8% of persons with PD had opioid prescription (Brefel‐Courbon et al. 2009).

Although the prevalence of pain across various stages of PD has been studied, the pharmacological treatment patterns have received less attention. The previous cross‐sectional studies are informative on the prevalence of opioid or analgesic use, which has been proposed as a proxy of pain (Brefel‐Courbon et al. 2009). However, longitudinal studies on opioid initiations are missing, although information on when opioids are initiated in terms of PD diagnosis would complement our understanding of the treatment of pain in PD. We investigated the incidence of opioid use in a nationwide cohort of persons with a PD diagnosis and compared the incidence to a matched cohort without PD 5 years before to 5 years after PD diagnosis.

## 2 Methods

The study was conducted within an exposure‐matched cohort study FINPARK, which includes 22,189 persons diagnosed with PD in Finland between 1996 and 2015 and their matched comparison persons without PD (*n* = 148,009). The derivation, inclusion and exclusion criteria of the FINPARK study have been described in more detail previously (Hentilä et al. 2021). Derivation of the study population for this study is described in Figure S1. People with PD diagnosis were identified from the Special reimbursement register maintained by the Social Insurance Institution of Finland, giving the study a nationwide coverage. The application for special reimbursement includes anamnesis of the patient and description of the characteristic clinical features of PD including bradykinesia, rigidity, and tremor. These applications are centrally reviewed at the Social Insurance Institution and special reimbursement for PD medications is granted if predefined criteria for PD diagnosis are fulfilled, and the diagnosis is confirmed by a neurologist. During the study period, the diagnosis of PD was consistent with United Kingdom Parkinson's Disease Society Brain Bank's criteria (Hughes et al. 1992).

To increase the sensitivity, we excluded people without a PD diagnosis ICD‐10 code for PD (G20) recorded in the Special reimbursement register (*n* = 1244), those who were < 35 years old at the time of PD diagnosis (*N* = 53) and those who had diagnoses whose symptoms may be confused with PD (*n* = 6456) within 2 years of PD diagnosis. These people were excluded, as the diagnosis of PD and its differential diagnostics is challenging, and false diagnoses are common in the early phase. The proportion of excluded people (25.9%) in our study is within the range of the estimated proportion of false diagnoses (Joutsa et al. 2014; Wermuth et al. 2015).

An age (±1 year), sex, and region matched comparison cohort was identified from a register of residents on the date of PD diagnosis. Thus, the comparison persons originate from the same source population as persons with PD. The comparison persons were not allowed to have dopaminergic PD drug purchases (Anatomical Therapeutic Chemical classification ATC code N04B) or reimbursement code for PD drugs ever before the index date, or 12 months after the index date (date of PD diagnosis; the matching date for the comparison persons). The exclusion criteria of the comparison cohort were otherwise identical to those applied in the PD cohort, but comparison persons with dementia due to PD (ICD‐10 F02.3) were also excluded.

This study was restricted to persons diagnosed in 2001–2014 (*N* = 16,340) and their 108,372 matched comparison persons to enable the assessment of opioid initiation for all study participants during the entire follow‐up. Initiations on opioid use (ATC code N02A) were captured from the Prescription register (1995–2019) during a 10‐year follow‐up (from 5 years before until 5 years after the index date). Initiation rates were calculated for any opioid use (N02A). Initiations were grouped into mild opioids (N02AA59 codeine, N02AJ06 codeine and paracetamol, N02AX02 tramadol, N02AJ13 tramadol and paracetamol, N02AC04 dextropropoxyphene and N02AD01 pentazocine), partial agonists (N02AE01 buprenorphine) and strong opioids (N02AA01 morphine, N02AA03 hydromorphone, N02AA05 oxycodone, N02AB03 fentanyl). Prevalent users with PD (*n* = 544) and without PD (*n* = 3209) were excluded with a 1‐year washout (Figure S1). As the Prescription register includes only dispensings from community pharmacies, we excluded persons who were hospitalised > 50% of the washout period or hospitalised for the last 90 days of the washout period. This study included 15,763 people who received an incident diagnosis of PD in 2001–2014. The matched comparison cohort without PD (*n* = 62,907) included up to four matched comparison persons for each person with PD identified. Altogether 99.2% had four comparison persons.

Information on sociodemographic characteristics, comorbidities, and medication use were retrieved from nationwide registers, including Care register for health care, Cancer register, Prescription register, and Special reimbursement register (Table S1). Data on medication use was obtained from the Prescription register from washout or 1 year before the initiation date. Usage of medication was defined with ATC codes (Table S1). Data on comorbidities since 1987 to the beginning of follow‐up/initiation date were obtained from the Special reimbursement register and Care register for health care, including epilepsy, asthma, or chronic obstructive pulmonary disease, cardiovascular diseases, and diabetes (Table S1). For the initiator‐noninitiator comparisons, the covariates are measured before the follow‐up. For the initiators with and without PD, covariates are measured before the initiation date.

## 3 Statistical Analyses

Differences in age (normal distribution) were evaluated with a *t*‐test, differences in time since the index date (skewed distribution) with a Mann–Whitney *U*‐test and in categorical variables with a chi‐squared test. These tests were performed to test differences between PD cases versus comparison persons, initiators vs. noninitiators, stratified by PD, and initiators with and without PD.

The incidence rates of opioid initiations per 100 person‐years were calculated in 6‐month time windows during the follow‐up. Those who were in hospital for 120 days or longer in a specific 6‐month window were excluded from that 6‐month period, because the Prescription register does not have information about medications used in hospitals. The follow‐up ended on opioid initiation, death, end of follow‐up (5 years from PD diagnosis) or end of data linkage (31.12.2019). The comparison people were censored if they were diagnosed with PD.

Incidence rate ratios comparing the persons with and without PD were calculated using Poisson regression. The matching characteristics (age, sex and region) were accounted for by using a clustered sandwich estimator for variance estimation. The statistical analyses were conducted with STATA MP 17.0.

### 3.1 Patient and Public Involvement in Research

This study used pseudonymized routinely collected healthcare register data and is a descriptive study of drug utilisation prevalence. Patients were not involved in study conceptualisation, design, conduct, or dissemination.

### 3.2 Ethical Permissions

The study was conducted under the Act on Secondary use of Health and Social Data. Because routinely collected, pseudonymized register data were used and study participants were not contacted, informed consent or separate ethic review by the institutional review board was not required. Permission for data use was obtained from the Finnish Social and Health Data Permit Authority Findata.

## 4 Results

### 4.1 Characteristics of PD and Comparison Cohort

This study included 15,763 people with PD and 62,907 people without PD (Table S2). The average age at index date was 70.8 years, and the majority (55.8%) were men. A history of psychiatric comorbidities and epilepsy was slightly more common in the PD cohort, and substance abuse was less common in the PD cohort at the beginning of follow‐up. However, the differences were minor (absolute difference ranging between 0.5% and 1.0%). The use of psychotropic drugs and analgesics other than opioids was more common in the PD cohort, but the differences were small. The largest difference was observed for antidepressants, with the prevalence of 8.2% in the PD cohort and 5.8% in the comparison cohort during the year preceding the beginning of follow‐up.

### 4.2 Rates of Opioid Initiation

**FIGURE 1:**
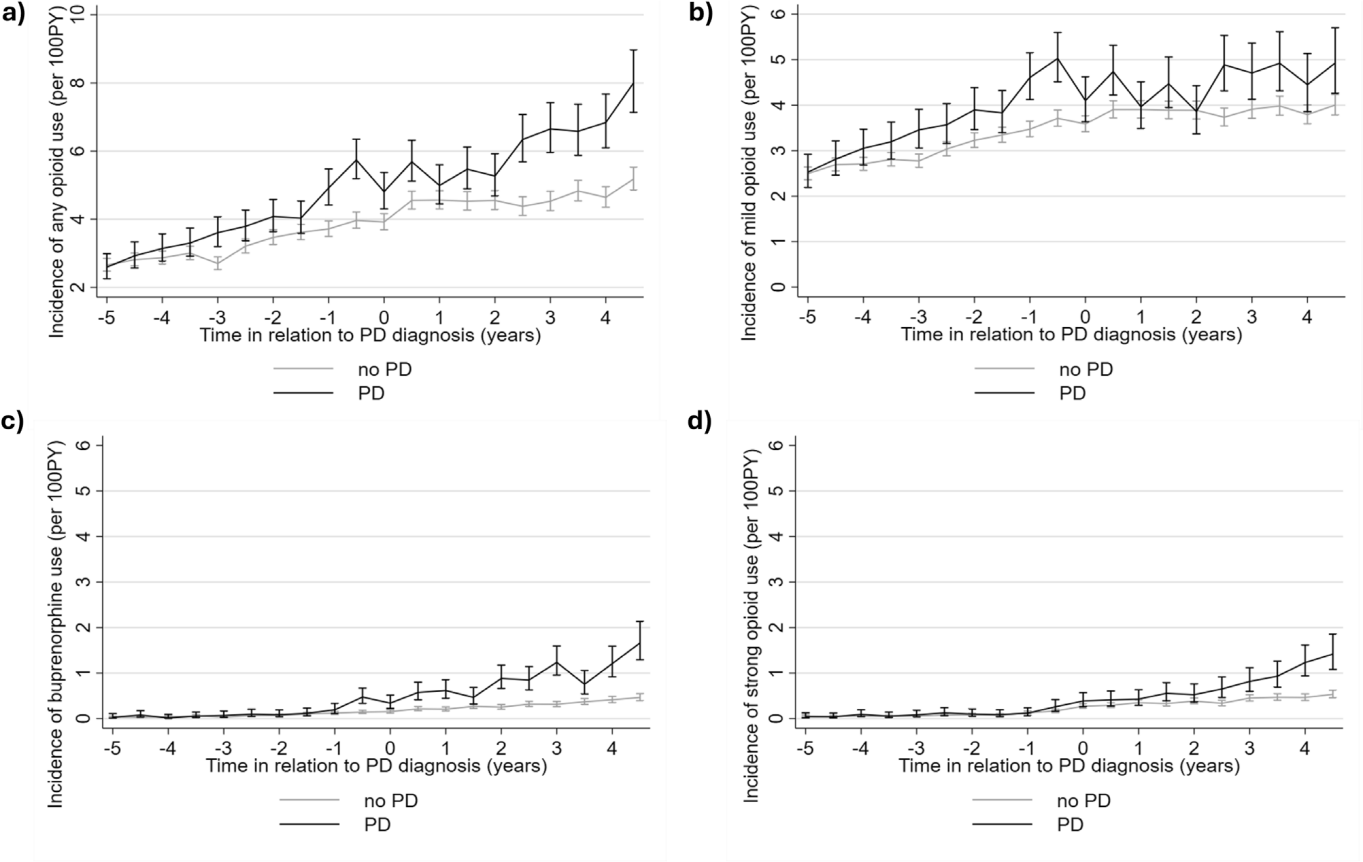
Incidence and 95% confidence intervals for (a) any opioid (b) mild opioid (c) buprenorphine (d) strong opioid use in relation to Parkinson's disease (PD) diagnosis date in PD and comparison cohorts (matched at the beginning of follow‐up for age, sex and region). The unadjusted rates were calculated per 100 person‐years (PY).

The rate of opioid initiations increased in both cohorts during the study period, and the initiation rate of opioids was higher in persons with PD from 3 years before the PD diagnosis onwards (Figure 1a and Table S3). The incidence was higher for all opioid categories (mild, buprenorphine and strong opioids), although the differences within the subcategories were smaller than for any opioid use (Figure 1b–d). The largest difference in mild opioid use (IRR 1.37, 95% CI 1.21–1.51) was observed 6 months prior to the diagnosis date (Table S3).

The incidence of buprenorphine initiation started to increase 6 months before PD diagnosis (IRR 3.09, 95% CI1.94–4.93) (Table S3). There was an increase in initiation rates of strong opioids after PD diagnosis, with the largest difference in initiations observed 4 years after PD diagnosis (IRR 2.47, 95% CI 1.76–3.45) (Table S3).

### 4.3 Characteristics of Opioid Initiators and Noninitiators With and Without PD

During the study period, 37.0% of persons with PD and 31.2% of persons without PD initiated opioids (Table 1). Opioid initiators were on average approximately 1 year older on the index date than noninitiators in both groups. The prevalence of comorbidities at the beginning of follow‐up was typically higher among initiators than noninitiators in both groups, although the prevalence differences were small. However, there was no significant difference in cancer prevalence between initiators and noninitiators in the PD cohort. The use of other analgesics, gabapentinoids, and psychotropics, except for antipsychotics, was more common among opioid initiators.

**TABLE 1:** The characteristics of opioid initiators and noninitiators with and without PD. Data are given as *n* (%) unless otherwise indicated.

	Parkinson's disease *N* = 15,763		*p*‐value	No Parkinson's disease *N* = 62,907		*p*‐value
	Initiators *N* = 5825 (37.0%)	Non‐initiators *N* = 9938 (63.0%)		Initiators *N* = 19,628 (31.2%)	Non‐initiators *N* = 43,279 (68.8%)	
Age at the index date, mean (95% CI)	71.6 (71.4–71.9)	70.4 (70.2–70.6)	< 0.001	71.7 (71.6–71.8)	70.4 (70.3–70.5)	< 0.001
Sex						
Women	2914 (50.0)	4049 (40.7)	< 0.001	8888 (45.3)	18,875 (43.6)	< 0.001
Men	2911 (50.0)	5889 (59.3)		10,740 (54.7)	24,404 (56.4)	
Occupational social class^a^						
Self‐employed	1542 (26.5)	2652 (26.6)	< 0.001	11,249 (26.0)	5152 (26.2)	< 0.001
Upper‐level employees	1064 (18.3)	2050 (20.6)		7494 (17.3)	3220 (16.4)	
Lower‐level employees	1482 (25.4)	2233 (22.5)		9871 (22.8)	4710 (24.0)	
Manual workers	1503 (25.8)	2562 (25.8)		12,038 (27.8)	5667 (28.9)	
Pensioners	195 (3.3)	355 (3.6)		1559 (3.6)	728 (3.7)	
Others	39 (0.7)	86 (0.9)		1068 (2.5)	151 (0.8)	
Comorbidities^a^						
Schizophrenia	63 (1.1)	137 (1.4)	0.108	155 (0.8)	402 (0.9)	0.084
Bipolar disorder or mania	54 (0.9)	65 (0.7)	0.056	96 (0.5)	133 (0.3)	< 0.001
Mood disorder (other than bipolar/mania)	255 (4.4)	261 (2.6)	< 0.001	621 (3.2)	821 (1.9)	< 0.001
Epilepsy	74 (1.3)	131 (1.3)	0.798	225 (1.1)	376 (0.9)	0.001
Asthma or chronic obstructive pulmonary disease	433 (7.4)	505 (5.1)	< 0.001	1664 (8.5)	2140 (4.9)	< 0.001
Cardiovascular diseases	2231 (38.3)	3159 (31.8)	< 0.001	7724 (39.4)	13,283 (30.7)	< 0.001
Stroke	211 (3.6)	256 (2.6)	< 0.001	828 (4.2)	1265 (2.9)	< 0.001
Diabetes	556 (9.5)	714 (7.2)	< 0.001	1980 (10.1)	2831 (6.5)	< 0.001
Cancer	101 (1.7)	144 (1.5)	0.163	349 (1.8)	504 (1.2)	< 0.001
Substance abuse	106 (1.8)	143 (1.4)	0.064	568 (2.9)	824 (1.9)	< 0.001
Medications^b^						
Any antidepressant	637 (10.9)	655 (6.6)	< 0.001	1589 (8.1)	2043 (4.7)	< 0.001
Duloxetine/Venlafaxine	41 (0.7)	41 (0.4)	0.014	98 (0.5)	86 (0.2)	< 0.001
Tricyclic antidepressants	158 (2.7)	154 (1.5)	< 0.001	430 (2.2)	504 (1.2)	< 0.001
Antipsychotics	205 (3.5)	346 (3.5)	0.901	384 (2.0)	815 (1.9)	0.534
Benzodiazepines and related drugs	1077 (18.5)	1158 (11.7)	< 0.001	3350 (17.1)	4378 (10.1)	< 0.001
Gabapentinoids	62 (1.1)	21 (0.2)	< 0.001	156 (0.8)	72 (0.2)	< 0.001
Paracetamol	196 (3.4)	105 (1.1)	< 0.001	657 (3.3)	506 (1.2)	< 0.001
Non‐steroidal anti‐inflammatory drugs	1969 (33.8)	2021 (20.3)	< 0.001	6787 (34.6)	8425 (19.5)	< 0.001

^a^ 1987‐before the follow‐up.

^b^ During washout (year before the follow‐up).

There was no difference in the age of opioid initiation between persons with and without PD (Table 2). The prevalence of somatic comorbidities was lower, and the prevalence of psychiatric and neurological disorders was higher among opioid initiators with PD. The prevalence of any antidepressant, tricyclic antidepressants (TCAs), duloxetine/venlafaxine, and gabapentinoids was also higher among opioid initiators with PD compared to those without PD. Opioid initiators with PD also had a higher prevalence of antipsychotic and benzodiazepine and related drug use than opioid initiators without PD.

**TABLE 2:** The characteristics of opioid initiators with and without PD. Data are given as *n* (%) unless otherwise indicated.

	Initiators with PD, *N* = 5825	Initiators without PD, *N* = 19,628	*p*‐value
Age at opioid initiation, mean (95% CI)	71.9 (71.6–72.1)	71.8 (71.7–72.0)	0.867
Sex			
Women	2914 (50.0)	8888 (45.3)	< 0.001
Men	2911 (50.0)	10,740 (54.7)	
Occupational social class^a^			
Self‐employed	1542 (26.5)	5152 (26.2)	< 0.001
Upper‐level employees	1064 (18.3)	3220 (16.4)	
Lower‐level employees	1482 (25.4)	4710 (24.0)	
Manual workers	1503 (25.8)	5667 (28.9)	
Pensioners	195 (3.3)	728 (3.7)	
Others	39 (0.7)	151 (0.8)	
Years since index date, median (interquartile range)	0.26 (−2.14 to 2.51)	0.25 (−2.16 to 2.48)	0.02
Comorbidities^b^			
Schizophrenia	101 (1.7)	203 (1.0)	< 0.001
Bipolar disorder or mania	65 (1.1)	117 (0.6)	< 0.001
Mood disorder (other than bipolar/mania)	447 (7.7)	849 (4.3)	< 0.001
Epilepsy	111 (1.9)	288 (1.5)	0.017
Asthma or chronic obstructive pulmonary disease	546 (9.4)	2145 (10.9)	0.001
Cardiovascular diseases	2541 (43.6)	8916 (45.4)	0.021
Stroke	479 (8.2)	1595 (8.1)	0.779
Diabetes	904 (15.5)	3254 (16.6)	0.063
Cancer	291 (5.0)	1427 (7.3)	< 0.001
Substance abuse	163 (2.8)	827 (4.2)	< 0.001
Medications^c^			
Any antidepressant	1227 (21.1)	2263 (11.5)	< 0.001
Duloxetine/Venlafaxine	119 (2.0)	235 (1.2)	< 0.001
Tricyclic antidepressants	171 (2.9)	438 (2.2)	0.002
Antipsychotics	415 (7.1)	600 (3.1)	< 0.001
Benzodiazepines and related drugs	1578 (27.1)	4670 (23.8)	< 0.001
Gabapentinoids	365 (6.3)	766 (3.9)	< 0.001
Paracetamol	1933 (33.2)	5677 (28.9)	< 0.001
Nonsteroidal anti‐inflammatory drugs	3150 (54.1)	10,592 (54.0)	0.757

^a^ 1987‐before the follow‐up.

^b^ 1987‐opioid initiation.

^c^ Year preceding the opioid initiation.

### 4.4 Characteristics of Initiators According to Type of Initiated Opioid

Among the opioid initiators with PD, the initiators of mild opioids were younger and had begun the opioid use before PD diagnosis (Table 3). Buprenorphine initiators were older and were more likely to be women. The median time of buprenorphine initiation was 2.5 years after PD diagnosis. The use of other analgesics, antidepressants, and antipsychotics was also most common in buprenorphine initiators, whereas the use of NSAIDs was most common among mild opioid initiators. In general, similar differences between initiators of different opioid classes were observed in persons without PD (Table S4). The prevalence of cancer was highest in the strong opioid initiators in both persons with and without PD (Table 3; Table S4).

**TABLE 3:** The characteristics of mild/buprenorphine/strong opioid initiators with PD. Data are given as *n* (%) unless otherwise indicated.

	Mild, *N* = 4899	Buprenorphine, *N* = 498	Strong, *N* = 428	*p*‐value
Age at opioid initiation (95% CI)	70.6 (70.4–70.9)	79.1 (78.4–79.8)	77.8 (77.0–78.6)	< 0.001
Sex				
Women	2391 (48.8)	300 (60.2)	223 (52.1)	< 0.001
Men	2508 (51.2)	198 (39.8)	205 (47.9)	
Occupational social class^a^				
Self‐employed	1296 (26.5)	142 (28.5)	104 (24.3)	0.002
Upper‐level employees	933 (19.0)	67 (13.5)	64 (15.0)	
Lower‐level employees	1241 (25.3)	132 (26.5)	109 (25.5)	
Manual workers	1247 (25.5)	134 (26.9)	122 (28.5)	
Pensioners	150 (3.1)	18 (3.6)	27 (6.3)	
Others	32 (0.7)	5 (1.0)	2 (0.5)	
Time since index date in years, median (interquartile range)	−0.21 (−2.36 to 2.13)	2.50 (0.79 to 3.85)	2.60 (0.75 to 4.02)	< 0.001
Comorbidities^b^				
Schizophrenia	64 (1.3)	24 (4.8)	13 (3.0)	< 0.001
Bipolar disorder or mania	58 (1.2)	2 (0.4)	5 (1.2)	0.285
Mood disorder (other than bipolar/mania)	341 (7.0)	57 (11.4)	49 (11.4)	< 0.001
Epilepsy	82 (1.7)	17 (3.4)	12 (2.8)	0.009
Asthma or chronic obstructive pulmonary disease	462 (9.4)	44 (8.8)	40 (9.3)	0.915
Cardiovascular diseases	2069 (42.2)	250 (50.2)	222 (51.9)	< 0.001
Stroke	340 (6.9)	64 (12.9)	75 (17.5)	< 0.001
Diabetes	733 (15.0)	96 (19.3)	75 (17.5)	0.019
Cancer	201 (4.1)	17 (3.4)	73 (17.1)	< 0.001
Substance abuse	135 (2.8)	14 (2.8)	14 (3.3)	0.827
Medications^c^				
Any antidepressant	943 (19.2)	167 (33.5)	117 (27.3)	< 0.001
Duloxetine/Venlafaxine	87 (1.8)	25 (5.0)	7 (1.6)	< 0.001
Tricyclic antidepressants	157 (3.2)	5 (1.0)	9 (2.1)	0.012
Antipsychotic	276 (5.6)	84 (16.9)	55 (12.9)	< 0.001
Benzodiazepines and related drugs	1295 (26.4)	143 (28.7)	140 (32.7)	0.014
Gabapentinoids	267 (5.5)	47 (9.4)	51 (11.9)	< 0.001
Paracetamol	1403 (28.6)	335 (67.3)	195 (45.6)	< 0.001
Non‐steroidal anti‐inflammatory drugs	2832 (57.8)	174 (34.9)	144 (33.6)	< 0.001

^a^ 1987‐before the follow‐up.

^b^ 1987‐opioid initiation.

^c^ Year preceding the opioid initiation.

## 5 Discussion

Pain is an important prodromal symptom of PD, which in our study was observed as the increasing incidence of any opioid use before PD diagnosis. In comparison to a matched cohort without PD, the initiation of opioids was more common in people with PD already before the PD diagnosis. The difference in initiations of any opioids and mild opioids emerged 3 years before the diagnosis (IRR 1.33). Initiations of buprenorphine started to increase 6 months before diagnosis whereas the difference in strong opioid initiations appeared after the diagnosis.

Our findings are in line with the higher prevalence of pain in persons with PD than in the general population (Nègre‐Pagès et al. 2008; Tai and Lin 2020), a cross‐sectional study showing a higher prevalence of opioids in persons with PD (Brefel‐Courbon et al. 2009), a higher prevalence of pain already before the PD diagnosis compared to a control group without PD (Bohlken et al. 2022; Schrag et al. 2015; Simonet et al. 2022) and the notion that chronic pain may begin already years before the PD clinical onset (Defazio et al. 2008). The increase in opioid initiation in this study occurred in the same time window as the incidence of muscle relaxants in the FINPARK study (Paakinaho et al. 2020), although a smaller difference to the comparison group was observed for opioids than muscle relaxants. Interestingly, the initiation rate of any opioid use and mild opioids appeared to stabilise around the time of diagnosis, which may reflect an improvement in pain when the PD treatment is initiated.

During the follow‐up, the initiation rates of opioids increased also in the comparison cohort, which likely reflects ageing of the population (Finnish Medicines Agency Fimea, and, Social Insurance Institution 2020). The study period covers the period of the worldwide increase in opioid prescriptions and the beginning of the opioid pandemic. However, in Finland, the non‐medical use of opioids has been less common than in for example, United States (Ollgren et al. 2014; Substance Abuse and Mental Health Services Administration 2013). According to a Finnish national study of medicolegal autopsies in Finland during 2010–2011, opioids were involved in 0.5% of these deaths. However, abuse in those aged 60 years or older was rare. (Häkkinen et al. 2014).

In our study, the incidence rates of all opioids remained above the comparison cohort after the diagnosis. Buprenorphine and strong opioids were more commonly initiated after PD diagnosis, which is in line with increased prevalence and intensity of pain in PD. As PD progresses, pain severity increases among persons with PD, which may result from increased rigidity (Valkovic et al. 2015), or higher dopaminergic dose as the disease progresses (Raja et al. 2020). However, it should be noted that pain in PD is more complex than “musculogenic” (de Andrade et al. 2023). Furthermore, different pain types are experienced among PD persons at advanced stages in comparison to those who are at early stages (Valkovic et al. 2015).

Several factors influence pain management, including pain intensity, age, and comorbidities. Opioids may also be prescribed alone or in combination with muscle relaxants for musculoskeletal pain (Larochelle et al. 2015). According to the Finnish Current Care Guideline of Pain management 2017, the first‐line medications for neuropathic pain include gabapentinoids, TCAs, and serotonin‐norepinephrine reuptake inhibitors including venlafaxine and duloxetine (Working group set up by the Finnish Medical Society Duodecim, The Finnish Society of Anaesthesiologists and the Finnish Association for General Practice 2017). In our study, the prevalence of these medications was higher in initiators with PD than opioid initiators without PD, which suggests a higher prevalence of neuropathic pain among them, although we could not assess different pain types. Higher prevalence of antidepressant use in opioid initiators compared with noninitiators could also reflect the impact of pain on mood. Based on previous studies, PD patients with pain have higher levels of depressive symptoms and anxiety than PD patients without pain (Defazio et al. 2008; Ehrt et al. 2009; Nègre‐Pagès et al. 2008).

Opioids have several adverse effects, including sedation, constipation, and hallucinations (El‐Tallawy et al. 2021). They also have the potential to escalate respiratory depression (Hsu et al. 2019). Therefore, their use requires careful monitoring, particularly in a population with elevated fall risk (Pelicioni et al. 2019). Risk of falling is a common problem for persons with PD; 68% of persons with PD experience falls at least once and 51% at least twice in 1 year (Wood et al. 2002). Falls may occur due to muscle weakness and balance/stability problems in PD (Montero‐Odasso et al. 2022) and may be further elevated by central nervous system‐acting drugs including antidepressants (Seppala et al. (2018)), especially TCAs (Seppala et al. 2021) and antiepileptic drugs such as gabapentinoids (Seppala et al. (2018)).

A previous US study observed an increase in opioid prescribing for chronic musculoskeletal pain, as well as increased benzodiazepine co‐prescription with opioid between 2001 and 2010 (Larochelle et al. 2015). The concomitant use of opioids and benzodiazepines should be avoided if possible. In our study, opioid initiators with PD had also used other fall‐risk increasing medications, including psychotropic medications and gabapentinoids. Substance use disorder, that is, dependency on prescription medication such as opioids, is prevalent among persons with PD (Kaur et al. 2021), but in our study there was no significant difference in the prevalence of substance abuse between opioid initiators and noninitiators with PD.

It should be noted that the scope of our study was to assess the opioid initiation rates in the PD and comparison cohort, and the indications for opioid use were not available. Therefore, the higher initiation rate in the PD cohort may be explained by a higher rate of falls and related injuries, chronic pain or pain related to PD. Indications and type of opioid‐treated pain in PD should be investigated in future studies. We were not able to investigate chronic pain because the diagnosis codes for chronic pain are severely underrecorded in these kinds of data sources, which more typically record the diagnosis codes for comorbidities causing chronic pain. Chronic comorbidities were more common in opioid initiators than noninitiators in our study, in line with previous observations on medical conditions predisposing to pain being more common in PD patients with pain compared to those without pain (Defazio et al. 2017). It should be noted that opioids are not the first‐line treatment of chronic pain according to the Finnish current care guidelines, and the inefficacy of opioids for different types of chronic pain has been demonstrated (Brefel‐Courbon et al. 2024; Chaparro et al. 2013; Noble et al. 2010). Cancer pain is an important indication of opioids (Chen et al. 2022), but in this study, the prevalence of cancer among opioid initiators was relatively low, and less common in initiators with PD than without PD, suggesting that the opioids were also used for treating non‐cancer pain. As our aim was to describe the opioid initiation rates regardless of indications, persons with cancer were not excluded from the study. One possible indication in PD is restless legs syndrome, affecting 15% of persons with PD (Trenkwalder, Winkelmann, et al. 2015; Yang et al. 2018).

We had no data on PD severity or fluctuations or on the duration of use or the dosage of prescribed opioids or dopaminergic medications used to treat PD. This study was restricted to prescribed opioids and thus does not cover non‐medical use. Further, the Prescription register covers reimbursed prescriptions and therefore the study may underestimate the prevalence. Similarly, the use of paracetamol as well as some NSAIDs including ibuprofen, acetylsalicylic acid (ASA), and naproxen is likely underestimated in our study, as these drugs are also available in low doses with small packages without a prescription. Nevertheless, the longitudinal assessment of opioid initiations in a large representative nationwide cohort provides information on the incidence of pain, as well as the clinical use of opioid agonists in PD. However, further studies assessing the prevalence of opioids, other analgesics, TCAs, and gabapentinoids used to treat neuropathic pain, as well as nonpharmacological treatment of pain in PD, are needed to understand the complexity of pain treatment and possible off‐label use of opioids in PD. Similarly, the treatment outcomes such as hospitalisations or long‐term health problems due to opioids in this population should be investigated.

In conclusion, the increased initiation rate of opioids is an early phenomenon in PD, manifesting already at the premotor or early motor stage, before the diagnosis is confirmed. This is likely predominantly driven by the management of pain, a common non‐motor symptom over the course of PD. As the disease progresses, resulting in increased disability and motor/non‐motor complications, there is a concomitant increase in the utilisation of opioids in PD patients. Further studies are warranted to investigate optimised balanced pharmacological strategies for pain management in PD. This information is relevant particularly in light of the undesirable adverse effects and events of opioids, which pose risks for PD patients susceptible to substance use disorders and a high risk for falls.

## Author Contributions

M.A.‐S.: Conceptualisation, Methodology, formal analysis, investigation, writing – original draft, writing – review and editing, visualisation. N.K.: Conceptualisation, Methodology, writing – original draft, writing – review and editing. A.P.: Methodology, investigation, writing – review and editing. M.K.: Methodology, investigation, writing – review and editing. V.K.: Writing – review and editing. S.H.: Conceptualisation, methodology, writing – review and editing. M.T.: Conceptualisation, methodology, writing – original draft, writing – review and editing. A.‐M.T.: Conceptualisation, methodology, formal analysis, investigation, data curation, writing – original draft, visualisation, supervision, writing – review and editing.

## Supporting information


Data S1.

## References

[ejp70056-bib-0001] Bohlken, J. , A. Schrag , S. Riedel‐Heller , and K. Kostev . 2022. “Identification of Prodromal Presentations of Parkinson's Disease Among Primary Care Outpatients in Germany.” Neuroepidemiology 56: 41–49. 34724667 10.1159/000520574

[ejp70056-bib-0002] Brefel‐Courbon, C. , S. Grolleau , C. Thalamas , et al. 2009. “Comparison of Chronic Analgesic Drugs Prevalence in Parkinson's Disease, Other Chronic Diseases and the General Population.” Pain 141: 14–18. 19062167 10.1016/j.pain.2008.04.026

[ejp70056-bib-0003] Brefel‐Courbon, C. , E. Harroch , A. Marques , et al. 2024. “Oxycodone or Higher Dose of Levodopa for the Treatment of Parkinsonian Central Pain: OXYDOPA Trial.” Movement Disorders 39: 1533–1543. 38850081 10.1002/mds.29878

[ejp70056-bib-0004] Broen, M. P. G. , M. M. Braaksma , J. Patijn , and W. E. J. Weber . 2012. “Prevalence of Pain in Parkinson's Disease: A Systematic Review Using the Modified QUADAS Tool.” Movement Disorders 27: 480–484. 22231908 10.1002/mds.24054

[ejp70056-bib-0005] Buhmann, C. , J. Kassubek , and W. H. Jost . 2020. “Management of Pain in Parkinson's Disease.” Journal of Parkinson's Disease 10: S37–S48. 10.3233/JPD-202069 PMC7592654 32568113

[ejp70056-bib-0006] Buhmann, C. , N. Wrobel , W. Grashorn , et al. 2017. “Pain in Parkinson Disease: A Cross‐Sectional Survey of Its Prevalence, Specifics, and Therapy.” Journal of Neurology 264: 758–769. 28243753 10.1007/s00415-017-8426-y

[ejp70056-bib-0007] Chaparro, L. E. , A. D. Furlan , A. Deshpande , A. Mailis‐Gagnon , S. Atlas , and D. C. Turk . 2013. “Opioids Compared to Placebo or Other Treatments for Chronic Low‐Back Pain.” Cochrane Database of Systematic Reviews 2013: CD004959. 23983011 10.1002/14651858.CD004959.pub4 PMC11056234

[ejp70056-bib-0008] Chen, Y. , S. Spillane , M. S. Shiels , et al. 2022. “Trends in Opioid Use Among Cancer Patients in the United States: 2013–2018.” JNCI Cancer Spectrum 6: pkab095. 35098020 10.1093/jncics/pkab095 PMC8793171

[ejp70056-bib-0009] de Andrade, D. C. , V. Mylius , S. Perez‐Lloret , et al. 2023. “Pain in Parkinson Disease: Mechanistic Substrates, Main Classification Systems, and How to Make Sense out of Them.” Pain 164: 2425–2434. 37318012 10.1097/j.pain.0000000000002968

[ejp70056-bib-0010] Defazio, G. , A. Antonini , M. Tinazzi , et al. 2017. “Relationship Between Pain and Motor and Non‐Motor Symptoms in Parkinson's Disease.” European Journal of Neurology 24: 974–980. 28516474 10.1111/ene.13323

[ejp70056-bib-0011] Defazio, G. , A. Berardelli , G. Fabbrini , et al. 2008. “Pain as a Nonmotor Symptom of Parkinson Disease: Evidence From a Case‐Control Study.” Archives of Neurology 65: 1191–1194. 18779422 10.1001/archneurol.2008.2

[ejp70056-bib-0012] Edinoff, A. , N. Sathivadivel , T. McBride , et al. 2020. “Chronic Pain Treatment Strategies in Parkinson's Disease.” Neurology International 12: 61–76. 33218135 10.3390/neurolint12030014 PMC7768530

[ejp70056-bib-0013] Ehrt, U. , J. P. Larsen , and D. Aarsland . 2009. “Pain and Its Relationship to Depression in Parkinson Disease.” American Journal of Geriatric Psychiatry 17: 269–275. 10.1097/jgp.0b013e31818af7ef 19322934

[ejp70056-bib-0014] El‐Tallawy, S. N. , R. Nalamasu , G. I. Salem , J. A. K. LeQuang , J. V. Pergolizzi , and P. J. Christo . 2021. “Management of Musculoskeletal Pain: An Update With Emphasis on Chronic Musculoskeletal Pain.” Pain and Therapy 10: 181–209. 33575952 10.1007/s40122-021-00235-2 PMC8119532

[ejp70056-bib-0015] Finnish Medicines Agency Fimea, and, Social Insurance Institution . 2020. “Finnish Statistics on Medicines 2019.”

[ejp70056-bib-0016] Ford, B. 2010. “Pain in Parkinson's Disease.” Movement Disorders 25, no. Suppl 1: S98–S103. 20187254 10.1002/mds.22716

[ejp70056-bib-0017] Guerriero, F. 2017. “Guidance on Opioids Prescribing for the Management of Persistent Non‐Cancer Pain in Older Adults.” World Journal of Clinical Cases 5: 73–81. 28352631 10.12998/wjcc.v5.i3.73 PMC5352962

[ejp70056-bib-0018] Häkkinen, M. , E. Vuori , and I. Ojanperä . 2014. “Prescription Opioid Abuse Based on Representative Postmortem Toxicology.” Forensic Science International 245: 121–125. 25447184 10.1016/j.forsciint.2014.10.028

[ejp70056-bib-0019] Hentilä, E. , M. Tiihonen , H. Taipale , S. Hartikainen , and A.‐M. Tolppanen . 2021. “Incidence of Antidepressant Use Among Community Dwellers With and Without Parkinson's Disease ‐ a Nationwide Cohort Study.” BMC Geriatrics 21: 202. 33757451 10.1186/s12877-021-02145-6 PMC7986562

[ejp70056-bib-0020] Hsu, J. R. , H. Mir , M. K. Wally , R. B. Seymour , and Orthopaedic Trauma Association Musculoskeletal Pain Task Force . 2019. “Clinical Practice Guidelines for Pain Management in Acute Musculoskeletal Injury.” Journal of Orthopaedic Trauma 33: e158–e182. 30681429 10.1097/BOT.0000000000001430 PMC6485308

[ejp70056-bib-0021] Hughes, A. J. , S. E. Daniel , L. Kilford , and A. J. Lees . 1992. “Accuracy of Clinical Diagnosis of Idiopathic Parkinson's Disease: A Clinico‐Pathological Study of 100 Cases.” Journal of Neurology, Neurosurgery, and Psychiatry 55: 181–184. 1564476 10.1136/jnnp.55.3.181 PMC1014720

[ejp70056-bib-0022] Joutsa, J. , M. Gardberg , M. Röyttä , and V. Kaasinen . 2014. “Diagnostic Accuracy of Parkinsonism Syndromes by General Neurologists.” Parkinsonism & Related Disorders 20: 840–844. 24816002 10.1016/j.parkreldis.2014.04.019

[ejp70056-bib-0023] Kaur, J. , R. K. Sandhu , K. T. Kubra , et al. 2021. “Substance Use Disorders in Patients With Parkinson's Disease and Adverse Hospitalization Outcomes: A National Inpatient Study.” Cureus 13: e16033. 34336520 10.7759/cureus.16033 PMC8321420

[ejp70056-bib-0024] Larochelle, M. R. , F. Zhang , D. Ross‐Degnan , and J. F. Wharam . 2015. “Trends in Opioid Prescribing and Co‐Prescribing of Sedative Hypnotics for Acute and Chronic Musculoskeletal Pain: 2001‐2010.” Pharmacoepidemiology and Drug Safety 24: 885–892. 25906971 10.1002/pds.3776

[ejp70056-bib-0025] Lee, M. A. , R. W. Walker , T. J. Hildreth , and W. M. Prentice . 2006. “A Survey of Pain in Idiopathic Parkinson's Disease.” Journal of Pain and Symptom Management 32: 462–469. 17085272 10.1016/j.jpainsymman.2006.05.020

[ejp70056-bib-0026] Montero‐Odasso, M. , N. van der Velde , F. C. Martin , et al. 2022. “World Guidelines for Falls Prevention and Management for Older Adults: A Global Initiative.” Age and Ageing 51: afac205. 36178003 10.1093/ageing/afac205 PMC9523684

[ejp70056-bib-0027] Mylius, V. , J. C. Möller , S. Bohlhalter , D. Ciampi de Andrade , and S. Perez Lloret . 2021. “Diagnosis and Management of Pain in Parkinson's Disease: A New Approach.” Drugs & Aging 38: 559–577. 34224103 10.1007/s40266-021-00867-1

[ejp70056-bib-0028] Mylius, V. , S. Perez Lloret , R. G. Cury , et al. 2021. “The Parkinson Disease Pain Classification System: Results From an International Mechanism‐Based Classification Approach.” Pain 162: 1201–1210. 33044395 10.1097/j.pain.0000000000002107 PMC7977616

[ejp70056-bib-0029] Nègre‐Pagès, L. , W. Regragui , D. Bouhassira , H. Grandjean , O. Rascol , and DoPaMiP Study Group . 2008. “Chronic Pain in Parkinson's Disease: The Cross‐Sectional French DoPaMiP Survey.” Movement Disorders 23: 1361–1369. 18546344 10.1002/mds.22142

[ejp70056-bib-0030] Noble, M. , J. R. Treadwell , S. J. Tregear , et al. 2010. “Long‐Term Opioid Management for Chronic Noncancer Pain.” Cochrane Database of Systematic Reviews 2010: CD006605. 20091598 10.1002/14651858.CD006605.pub2 PMC6494200

[ejp70056-bib-0031] Ollgren, J. , M. Forsell , V. Varjonen , et al. 2014. “Amfetamiinien Ja Opioidien ongelmakäytön Yleisyys Suomessa 2012 [the Prevalence of Amphetamine and Opioid Abuse in Finland in 2012].” Yhteiskuntapolitiikka 5: 498–508.

[ejp70056-bib-0032] Paakinaho, A. , N. Karttunen , M. Koponen , et al. 2020. “Incidence of Muscle Relaxant Use in Relation to Diagnosis of Parkinson's Disease.” International Journal of Clinical Pharmacy 42: 336–340. 32144610 10.1007/s11096-020-01002-7

[ejp70056-bib-0033] Pelicioni, P. H. S. , J. C. Menant , M. D. Latt , and S. R. Lord . 2019. “Falls in Parkinson's Disease Subtypes: Risk Factors, Locations and Circumstances.” International Journal of Environmental Research and Public Health 16: 2216. 31234571 10.3390/ijerph16122216 PMC6616496

[ejp70056-bib-0034] Postuma, R. B. , D. Berg , M. Stern , et al. 2015. “MDS Clinical Diagnostic Criteria for Parkinson's Disease.” Movement Disorders 30: 1591–1601. 26474316 10.1002/mds.26424

[ejp70056-bib-0035] Raja, K. , S. Ramrakhia , K. Dev , et al. 2020. “The Risk Factors for the Wearing‐Off Phenomenon in Parkinson's Disease.” Cureus 12: e10729. 33145134 10.7759/cureus.10729 PMC7599057

[ejp70056-bib-0036] Schrag, A. , L. Horsfall , K. Walters , A. Noyce , and I. Petersen . 2015. “Prediagnostic Presentations of Parkinson's Disease in Primary Care: A Case‐Control Study.” Lancet Neurology 14: 57–64. 25435387 10.1016/S1474-4422(14)70287-X

[ejp70056-bib-0037] Seppala, L. J. , M. Petrovic , J. Ryg , et al. 2021. “STOPPFall (Screening Tool of Older Persons Prescriptions in Older Adults With High Fall Risk): A Delphi Study by the EuGMS Task and Finish Group on Fall‐Risk‐Increasing Drugs.” Age and Ageing 50: 1189–1199. 33349863 10.1093/ageing/afaa249 PMC8244563

[ejp70056-bib-0038] Seppala, L. J. , E. M. M. van de Glind , J. G. Daams , et al. 2018. “Fall‐Risk‐Increasing Drugs: A Systematic Review and Meta‐Analysis: III. Others.” Journal of the American Medical Directors Association 19: 372.e1. 10.1016/j.jamda.2017.12.099 29402646

[ejp70056-bib-0039] Seppala, L. J. , A. M. A. T. Wermelink , M. de Vries , et al. 2018. “Fall‐Risk‐Increasing Drugs: A Systematic Review and Meta‐Analysis: II. Psychotropics.” Journal of the American Medical Directors Association 19: 371.e11. 10.1016/j.jamda.2017.12.098 29402652

[ejp70056-bib-0040] Seppi, K. , K. Ray Chaudhuri , M. Coelho , et al. 2019. “Update on Treatments for Nonmotor Symptoms of Parkinson's Disease‐An Evidence‐Based Medicine Review.” Movement Disorders 34: 180–198. 30653247 10.1002/mds.27602 PMC6916382

[ejp70056-bib-0041] Simonet, C. , J. Bestwick , M. Jitlal , et al. 2022. “Assessment of Risk Factors and Early Presentations of Parkinson Disease in Primary Care in a Diverse UK Population.” JAMA Neurology 79: 359–369. 35254398 10.1001/jamaneurol.2022.0003 PMC8902684

[ejp70056-bib-0042] Sophie, M. , and B. Ford . 2012. “Management of Pain in Parkinson's Disease.” CNS Drugs 26: 937–948. 23055368 10.1007/s40263-012-0005-2

[ejp70056-bib-0043] Substance Abuse and Mental Health Services Administration . 2013. “Results From the 2012 National Survey on Drug Use and Health: Summary of National Findings. (Rockville, MD: Substance Abuse and Mental Health Services Administration).”

[ejp70056-bib-0044] Tai, Y.‐C. , and C.‐H. Lin . 2020. “An Overview of Pain in Parkinson's Disease.” Clinical Parkinsonism & Related Disorders 2: 1–8. 34316612 10.1016/j.prdoa.2019.11.004 PMC8302194

[ejp70056-bib-0045] Trenkwalder, C. , K. R. Chaudhuri , P. Martinez‐Martin , et al. 2015. “Prolonged‐Release Oxycodone‐Naloxone for Treatment of Severe Pain in Patients With Parkinson's Disease (PANDA): A Double‐Blind, Randomised, Placebo‐Controlled Trial.” Lancet Neurology 14: 1161–1170. 26494524 10.1016/S1474-4422(15)00243-4

[ejp70056-bib-0046] Trenkwalder, C. , J. Winkelmann , Y. Inoue , and W. Paulus . 2015. “Restless Legs Syndrome‐Current Therapies and Management of Augmentation.” Nature Reviews. Neurology 11: 434–445. 26215616 10.1038/nrneurol.2015.122

[ejp70056-bib-0047] Valkovic, P. , M. Minar , H. Singliarova , et al. 2015. “Pain in Parkinson's Disease: A Cross‐Sectional Study of Its Prevalence, Types, and Relationship to Depression and Quality of Life.” PLoS One 10: e0136541. 26309254 10.1371/journal.pone.0136541 PMC4550419

[ejp70056-bib-0048] Wermuth, L. , X. Cui , N. Greene , E. Schernhammer , and B. Ritz . 2015. “Medical Record Review to Differentiate Between Idiopathic Parkinson's Disease and Parkinsonism: A Danish Record Linkage Study With 10 Years of Follow‐Up.” Parkinsons Disease 2015: 781479. 10.1155/2015/781479 PMC4681800 26770868

[ejp70056-bib-0049] WHO . 2018. Guidelines for the Pharmacological and Radiotherapeutic Management of Cancer Pain in Adults and Adolescents. World Health Organization. 30776210

[ejp70056-bib-0050] Wood, B. H. , J. A. Bilclough , A. Bowron , and R. W. Walker . 2002. “Incidence and Prediction of Falls in Parkinson's Disease: A Prospective Multidisciplinary Study.” Journal of Neurology, Neurosurgery, and Psychiatry 72: 721–725. 12023412 10.1136/jnnp.72.6.721 PMC1737913

[ejp70056-bib-0051] Working group set up by the Finnish Medical Society Duodecim, The Finnish Society of Anaesthesiologists and the Finnish Association for General Practice . 2017. Current Care Guidelines: Pain (Helsinki, Finland: The Finnish Medical Society Duodecim).

[ejp70056-bib-0052] Yang, X. , B. Liu , H. Shen , et al. 2018. “Prevalence of Restless Legs Syndrome in Parkinson's Disease: A Systematic Review and Meta‐Analysis of Observational Studies.” Sleep Medicine 43: 40–46. 29482811 10.1016/j.sleep.2017.11.1146

