# Supplementary material for: Incidence of Opioid Use Before and After Parkinson's Disease Diagnosis
Source: Eur J Pain. 2025 Jun 9;29(6):e70056. doi: 10.1002/ejp.70056 (PMC12146810; doi:10.1002/ejp.70056)

# **Supplementary material**

# Al-Sagheer et al Incidence of opioid use before and after Parkinson’s disease diagnosis

## Supplementary table 1. Covariate definitions and data sources

| Covariate | Data sources & coding | Years |
| --- | --- | --- |
| Age | Population register, years | index date & initiation date |
| Sex | Population register, women/men | constant |
| Occupational social class | 1: Self-employed  2: Upper-level employees with administrative, managerial, professional and related occupations  3: Lower-level employees with administrative and clerical occupations  4: Manual workers  5: Pensioners  6: Others (Students, long-term unemployed, other positions not elsewhere classified, socioeconomic status unknown)  7: missing | before the follow-up |
| Time since index date | (years, negative values=before index date, positive values=after the index date) | initiation date |
| Comorbidities |  |  |
| Schizophrenia | Care register for health care  ICD-9: 295, 297, 298 (1987-1995)  ICD-10: F20-F29 (1996 onwards) | From 1987 to the beginning of follow-up/initiation date |
| Bipolar disorder or mania | Care register for health care  ICD-9: 2962, 2963, 2964, 2967 (1987-1995)  ICD-10: F30, F31 (1996 onwards) | From 1987 to the beginning of follow-up/initiation date |
| Mood disorder (other than bipolar/mania) | Care register for health care  ICD-9: 2961, 2968A, 3004A, 3011D (1987-1995)  ICD-10: F32-F39 (1996 onwards) | From 1987 to the beginning of follow-up/initiation date |
| Epilepsy | Special reimbursement register code 111 | From 1987 to the beginning of follow-up/initiation date |
| Asthma/chronic obstructive pulmonary disease | Special reimbursement register code 203 | From 1987 to the beginning of follow-up/initiation date |
| Cardiovascular diseases | Special reimbursement register code: chronic heart failure (201), chronic blood pressure (205), Chronic coronary artery disease and lipid metabolism disorder associated with chronic coronary artery disease (206), Chronic cardiac arrhythmias (207), dyslipidemia in chronic coronary artery disease (213), clopidogrel (280) | From 1987 to the beginning of follow-up/initiation date |
| Stroke (stroke) | Care register for health care  ICD-9: 430-432,4360,4330A,4331A,4339A,4340A, 4341A,4349A,4380A (1987-1995)  ICD-10 I60-I64, I69 (1996 onwards) | From 1987 to the beginning of follow-up/initiation date |
| Diabetes | Prescription register: ATC code A10 excluding A10BX01(guar gum)  Special reimbursement register code 103 | From 1995 to the beginning of follow-up/initiation date  From 1987 to the beginning of follow-up/initiation date |
| Cancer | Cancer register: International Agency for Research on Cancer (CRG: Collaborative Research Group) (IARC Tools) code C* | Within two years before the beginning of follow-up/initiation date |
| Substance abuse | Prescription register: ATC codes N07BB, N07BC  Care register for health care  Reason for admission: (33,71,72,73,74,75)  ICD-10 F1*, K860, K70, G621, G312, G721, I426, K292, R780 (1996 onwards)  ICD-9 291,292,303,304,305,3575, 3594A, 4255A,5353A,5770D-F,5771C,5710A,5711A, 5712A,5713X (1987-1995) | from 1995 to the beginning of follow-up/initiation date  From 1987 to the beginning of follow-up/initiation date |
| Any antidepressant | Prescription register: ATC code N06A | washout/One year before the initiation date |
| Duloxetine/venlafaxine | Prescription register: ATC code N06AX21, N06AX16 | washout/One year before the initiation date |
| Tricyclic antidepressants (TCAs) | Prescription register: ATC code N06AA | washout/One year before the initiation date |
| Antipsychotic | Prescription register: ATC code N05A  (excluding N05AN and N05AB04) | washout/One year before the initiation date |
| Benzodiazepines and related drugs | Prescription register: ATC code N05BA, N05CD, N05CF | washout/One year before the initiation date |
| Gabapentinoid | Prescription register: ATC code N03AX12, N03AX16 | washout/One year before the initiation date |
| Paracetamol | Prescription register: ATC code N02BE01, N02AJ06, N02AJ13 | washout/One year before the initiation date |
| Non-steroidal anti-inflammatory drugs (NSAIDs) | Prescription register: ATC code M01A | washout/One year before the initiation date |

## Supplementary Table 2. The characteristics of PD and comparison cohort. Data are given as n (%) unless otherwise indicated.

|  | Parkinson’s disease  N= 15,763 | Comparison cohort  N= 62,907 | p-value |
| --- | --- | --- | --- |
| Age at the index date,  mean (95 % Cl) | 70.8 (70.7-71.0) | 70.8 (70.7-70.9) | matched |
| Sex  Women  Men | 6963 (44.2)  8800 (55.8) | 27763 (44.1)  35144 (55.9) | matched |
| Occupational social class^a^  Self-employed  Upper-level employees  Lower-level employees  Manual workers  Pensioners  Others | 4194 (26.6)  3114 (19.8)  3715 (23.6)  4065 (25.8)  550 (3.5)  125 (0.8) | 16401 (26.1)  10714 (17.0)  14581 (23.2)  17705 (28.1)  2287 (3.6)  1219 (1.9) | <0.001 |
| Comorbidities^b^ |  |  |  |
| Schizophrenia | 200 (1.3) | 557 (0.9) | <0.001 |
| Bipolar disorder or mania | 119 (0.8) | 229 (0.4) | <0.001 |
| Mood disorder  (other than bipolar/mania) | 516 (3.3) | 1442 (2.3) | <0.001 |
| Epilepsy | 205 (1.3) | 601 (1.0) | <0.001 |
| Asthma or chronic obstructive pulmonary disease | 938 (6.0) | 3804 (6.0) | 0.65 |
| Cardiovascular diseases | 5390 (34.2) | 21007 (33.4) | 0.057 |
| Stroke | 467 (3.0) | 2093 (3.3) | 0.021 |
| Diabetes | 1270 (8.1) | 4811 (7.6) | 0.086 |
| Cancer | 245 (1.6) | 853 (1.4) | 0.058 |
| Substance abuse | 249 (1.6) | 1392 (2.2) | <0.001 |
| Medications^b^ |  |  |  |
| Any antidepressant | 1292 (8.2) | 3632 (5.8) | <0.001 |
| Duloxetine/Venlafaxine | 82 (0.5) | 184 (0.3) | <0.001 |
| Tricyclic antidepressants | 312 (2.0) | 934 (1.5) | <0.001 |
| Antipsychotics | 551 (3.5) | 1199 (1.9) | <0.001 |
| Benzodiazepines & related drugs | 2235 (14.2) | 7728 (12.3) | <0.001 |
| Gabapentinoids | 83 (0.5) | 228 (0.4) | 0.003 |
| Paracetamol | 301 (1.9) | 1163 (1.8) | 0.61 |
| Non-steroidal anti-inflammatory drugs | 3990 (25.3) | 15212 (24.2) | 0.003 |
| Reason for ending the follow-up | |  | <0.001 |
| End of data linkage | 7809 (49.5) | 37874 (60.2) |  |
| Death | 2129 (13.5) | 5372 (8.5) |  |
| Conversion to PD | not applicable | 33 (0.05) |  |
| Initiated opioid | 5825 (37.0) | 19268 (31.2) |  |

^a^1987-before the follow-up

^b^during washout (year before the follow-up)

Supplementary Table 3. Incidence rate ratios of any, mild, buprenorphine and strong opioid per 100 person-years with 95% confidence intervals.

| Time since index date  (in years) | Any opioid | Mild | Buprenorphine | Strong |
| --- | --- | --- | --- | --- |
| -5 | 0.98 (0.84-1.14) | 0.99 (0.84-1.15) | 0.89 (0.19-4.10) | 0.63 (0.19-2.13) |
| -4.5 | 1.04 (0.90-1.20) | 1.03 (0.89-1.19) | 2.39 (0.87-6.59) | 0.80 (0.23-2.76) |
| -4 | 1.10 (0.95-1.26) | 1.09 (0.95-1.26) | 0.40 (0.05-3.12) | 2.00 (0.81-4.95) |
| -3.5 | 1.10 (0.96-1.26) | 1.10 (0.95-1.26) | 0.89 (0.30-2.63) | 1.45 (0.46-4.57) |
| -3 | 1.33 (1.16-1.53) | 1.32 (1.14-1.52) | 2.00 (0.68-5.86) | 1.60 (0.62-4.13) |
| -2.5 | 1.18 (1.03-1.35) | 1.16 (1.01-1.33) | 1.34 (0.57-3.16) | 1.58 (0.73-3.40) |
| -2 | 1.17 (1.03-1.34) | 1.18 (1.03-1.35) | 1.01 (0.41-2.47) | 1.13 (0.49-2.61) |
| -1.5 | 1.11 (0.98-1.27) | 1.11 (0.97-1.28) | 1.20 (0.55-2.64) | 1.06 (0.43-2.60) |
| -1 | 1.32 (1.17-1.49) | 1.33 (1.17-1.51) | 1.65 (0.87-3.14) | 0.79 (0.37-1.69) |
| -0.5 | 1.44 (1.28-1.62) | 1.37 (1.21-1.55) | 3.09 (1.94-4.93) | 1.58 (0.90-2.76) |
| 0 | 1.22 (1.08-1.39) | 1.16 (1.01-1.33) | 2.67 (1.58-4.52) | 1.38 (0.88-2.15) |
| 0.5 | 1.24 (1.11-1.40) | 1.18 (1.04-1.34) | 2.14 (1.43-3.21) | 1.25 (0.80-1.96) |
| 1 | 1.09 (0.96-1.24) | 0.99 (0.86-1.13) | 2.80 (1.84-4.25) | 1.23 (0.79-1.93) |
| 1.5 | 1.20 (1.06-1.36) | 1.14 (1.00-1.31) | 1.55 (0.99-2.43) | 1.51 (1.00-2.27) |
| 2 | 1.15 (1.01-1.31) | 0.97 (0.83-1.13) | 3.66 (2.48-5.40) | 1.45 (0.95-2.21) |
| 2.5 | 1.43 (1.27-1.63) | 1.31 (1.13-1.50) | 2.49 (1.71-3.61) | 1.83 (1.22-2.75) |
| 3 | 1.45 (1.28-1.64) | 1.23 (1.07-1.43) | 3.90 (2.75-5.54) | 1.78 (1.23-2.58) |
| 3.5 | 1.35 (1.19-1.53) | 1.22 (1.05-1.41) | 1.93 (1.29-2.88) | 1.94 (1.35-2.79) |
| 4 | 1.45 (1.28-1.66) | 1.17 (1.00-1.37) | 2.79 (1.97-3.95) | 2.47 (1.76-3.45) |
| 4.5 | 1.52 (1.34-1.73) | 1.18 (1.00-1.39) | 3.21 (2.33-4.42) | 2.39 (1.72-3.34) |

##

## Supplementary Table 4. The characteristics of mild/buprenorphine/strong opioid initiators without PD.

|  | Mild  17,615 | Buprenorphine  881 | Strong  1,132 | P-value |
| --- | --- | --- | --- | --- |
| Age at initiation (95 % Cl) | 71.1 (70.9-71.2) | 79.1 (78.6-79.6) | 76.2 (75.7-76.7) | <0.001 |
| Sex        Women        Men | 10232 (45.4)  12282 (54.6) | 835 (60.6)  544 (39.4) | 735 (47.1)  825 (52.9) | <0.001 |
| Occupational social class^a^        Self-employed        Upper-level employees        Lower-level employees        Manual workers        Pensioners        Others | 5900 (26.2)  3905 (17.3)  5502 (24.4)  6318 (28.1)  733 (3.3)  156 (0.7) | 383 (27.8)  167 (12.1)  334 (24.2)  387 (28.1)  88 (6.4)  20 (1.5) | 411 (26.3)  212 (13.6)  356 (22.8)  465 (29.8)  102 (6.5)  14 (0.9) | <0.001 |
| Time since index date in years, median (interquartile range) | -.027 (-2.35 to 2.28) | 2.13 (0.29 to 3.69) | 2.02 (0.35 to 3.61) | <0.001 |
| Comorbidities^b^ |  |  |  |  |
| Schizophrenia | 222 (1.0) | 46 (3.3) | 36 (2.3) | <0.001 |
| Bipolar disorder or mania | 161 (0.7) | 10 (0.7) | 11 (0.7) | 0.459 |
| Mood disorder  (other than bipolar/mania) | 1088 (4.8) | 104 (7.5) | 104 (6.7) | 0.199 |
| Epilepsy | 315 (1.4) | 45 (3.3) | 39 (2.5) | <0.001 |
| Asthma or chronic obstructive pulmonary disease | 2360 (10.5) | 155 (11.2) | 176 (11.3) | 0.118 |
| Cardiovascular diseases | 9921 (44.1) | 742 (53.8) | 794 (50.9) | <0.001 |
| Stroke | 1637 (7.3) | 208 (15.1) | 229 (14.7) | <0.001 |
| Diabetes | 3569 (15.9) | 294 (21.3) | 295 (18.9) | <0.001 |
| Cancer | 1188 (5.3) | 73 (5.3) | 457 (29.3) | <0.001 |
| Substance abuse | 853 (3.8) | 61 (4.4) | 76 (4.9) | 0.018 |
| Medications^c^ |  |  |  |  |
| Any antidepressant | 2818 (12.5) | 360 (26.1) | 312 (20.0) | <0.001 |
| Duloxetine/Venlafaxine | 283 (1.3) | 47 (3.4) | 24 (1.5) | 0.001 |
| Tricyclic antidepressants | 552 (2.5) | 27 (2.0) | 30 (1.9) | 0.601 |
| Antipsychotic | 686 (3.0) | 198 (14.4) | 131 (8.4) | <0.001 |
| Benzodiazepines & related drugs | 5375 (23.9) | 415 (30.1) | 458 (29.4) | <0.001 |
| Gabapentinoids | 891 (4.0) | 106 (7.7) | 134 (8.6) | <0.001 |
| Paracetamol | 6033 (26.8) | 885 (64.2) | 692 (44.4) | <0.001 |
| Non-steroidal anti-inflammatory drugs | 12753 (56.6) | 444 (32.2) | 545 (34.9) | <0.001 |

^a^1987-before the follow-up

^b^1987-opioid initiation

^c^year preceding the opioid initiation

##

## Supplementary figure 1. Formation of study population. Details on exclusion criteria for 7753 excluded persons with PD are given in full detail in Hentilä et al BMC Geriatrics 2021 (reference 23 of the manuscript). In this study, the maximum number of matched comparison persons for each person with PD was restricted to four to avoid possible bias arising from uneven number of comparison persons after applying study-specific exclusion criteria.

##
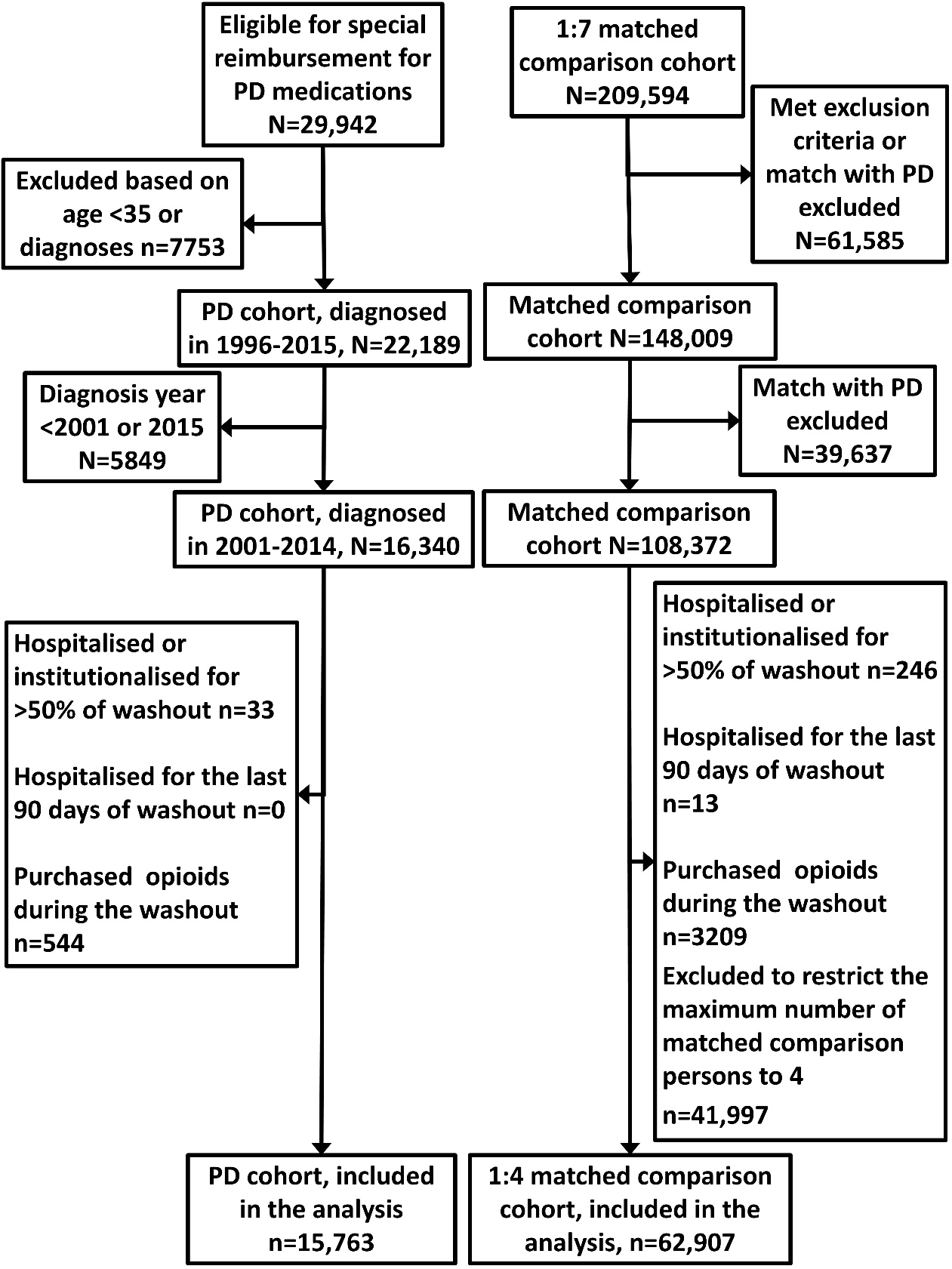

Supplement: Supplementary file 1 — Data S1. [file EJP-29-0-s001.docx]
